# Supplementary material for: Constraints on the martian crust away from the InSight landing site
Source: Nat Commun. 2022 Dec 26;13:7950. doi: 10.1038/s41467-022-35662-y (PMC9792460; doi:10.1038/s41467-022-35662-y)
Supplement: Supplementary file 1 — Supplementary Information [file 41467_2022_35662_MOESM1_ESM.pdf]

Supplementary Information

Constraints on the Martian Crust Away From the InSight Landing Site

Jiaqi Li[1*], Caroline Beghein[1], Scott M. McLennan[2], Anna C. Horleston[3], Constantinos Charalambous[4], Quancheng Huang[5], Géraldine Zenhäusern[6], Ebru Bozdag[5], W. T. Pike[4], Matthew Golombek[7], Ved Lekić[8], Philippe Lognonné[9], and W. Bruce Banerdt[7]

[1] Department of Earth, Planetary, and Space Sciences, University of California, Los Angeles, CA 90095, USA. E-mail: jli@epss.ucla.edu

[2] Department of Geosciences, Stony Brook University, Stony Brook, New York, 11794–2100 USA

[3] School of Earth Sciences, University of Bristol, Bristol, United Kingdom

[4] Department of Electrical and Electronic Engineering, Imperial College London, London, UK

[5] Department of Geophysics, Colorado School of Mines, Golden, CO, USA

[6] Institute of Geophysics, ETH Zurich, Zurich, Switzerland

[7] Jet Propulsion Laboratory, California Institute of Technology, Pasadena, CA 91109, USA.

[8] Department of Geology, University of Maryland, College Park, Maryland, USA.

[9] Université Paris Cité, Institut de physique du globe de Paris, CNRS, Paris, F-75005, France.

* Corresponding author: jli@epss.ucla.edu

Overview

This supplementary information contains 16 figures and one table.

List of Figures/Tables

Supplementary Figure 1. SS and PP phase picks.
Supplementary Figure 2. Raw waveforms on three components.
Supplementary Figure 3. Probability density functions for the differential arrival and amplitude ratio.
Supplementary Figure 4. Synthetic arrival and amplitude ratio for the receiver-function-derived models on Mars.
Supplementary Figure 5. Synthetic amplitude ratio from different discontinuities.
Supplementary Figure 6. Hyperparameters from other studies.
Supplementary Figure 7. Comodulation analysis.
Supplementary Figure 8. Searching for earlier precursors.
Supplementary Figure 9. Geological map near the bounce point.
Supplementary Figure 10. Inversion results for different density-to-Vs values.
Supplementary Figure 11. Benchmark of the forward modeling approaches
Supplementary Figure 12. Seismic velocity profiles from the inversion with updated priors.
Supplementary Figure 13. Inversion results based on the waveform misfit.
Supplementary Figure 14. Comodulation analysis for the PP precursor.
Supplementary Figure 15. Sensitivity kernel.
Supplementary Figure 16. Analysis of the porosity effects on the seismic wave velocities.
Supplementary Table 1. Elastic properties of the materials in the porosity simulation.

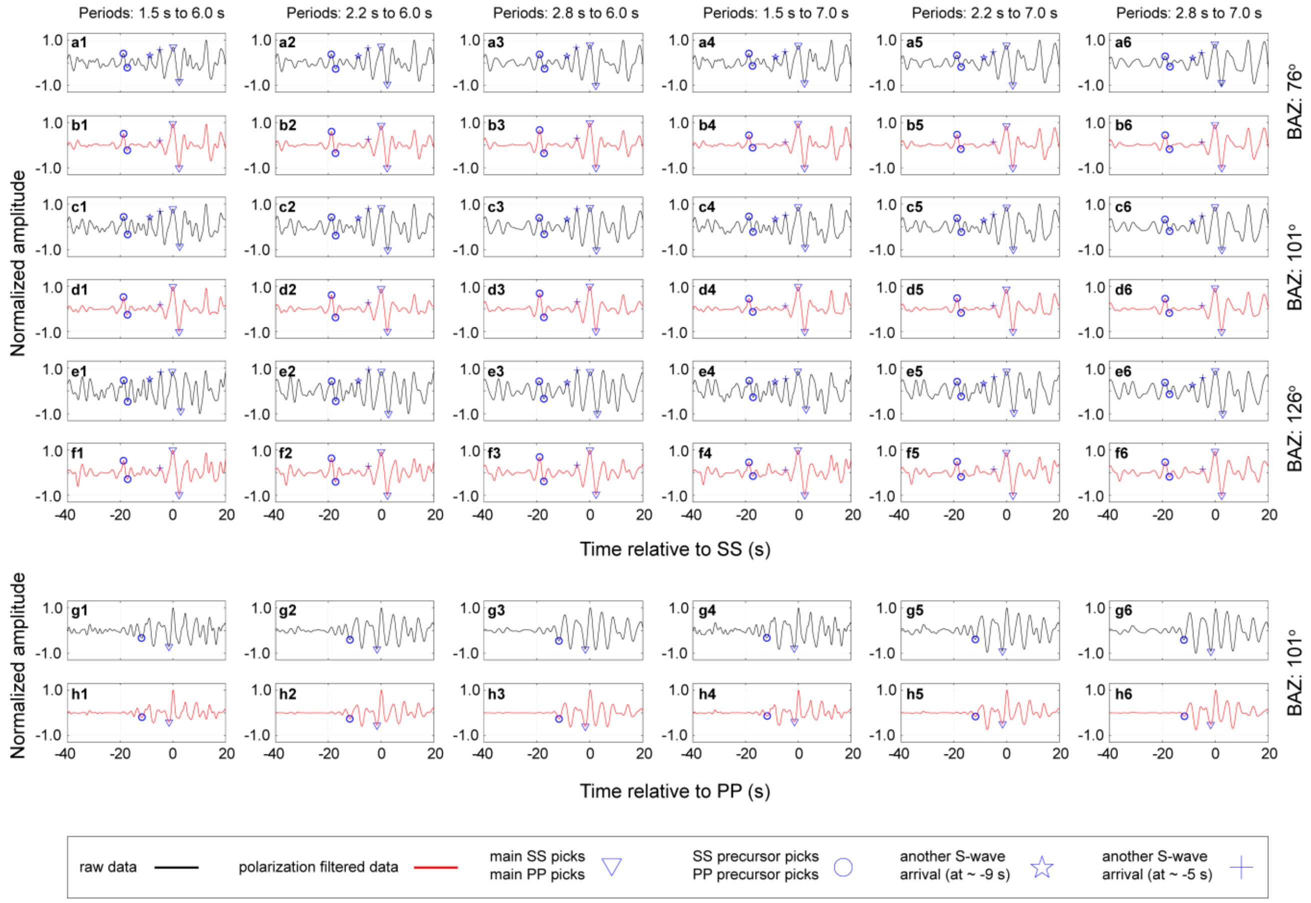

Supplementary Figure 1. SS and PP phase picks.

(a1-a6) SS and SS precursor phase picks for the raw displacement data on the tangential component (rotated based on a minimum back azimuth of 76 degrees) at six different frequency bands (i.e., 1.5 – 6.0 s, 2.2 – 6.0 s, 2.8 – 6.0 s, 1.5 – 7.0 s, 2.2 – 7.0 s, 2.8 – 7.0 s).

(b1-b6) similar to (a1-a6) but for the polarization-filtered data.

(c1-d6) similar to (a1-b6) but based on a back azimuth of 101 degrees.

(e1-f6) similar to (a1-b6) but based on a maximum back azimuth of 126 degrees.

(g1-h6) similar to (a1-b6) but for the PP and PP precursor on the vertical component (independent of back azimuth).

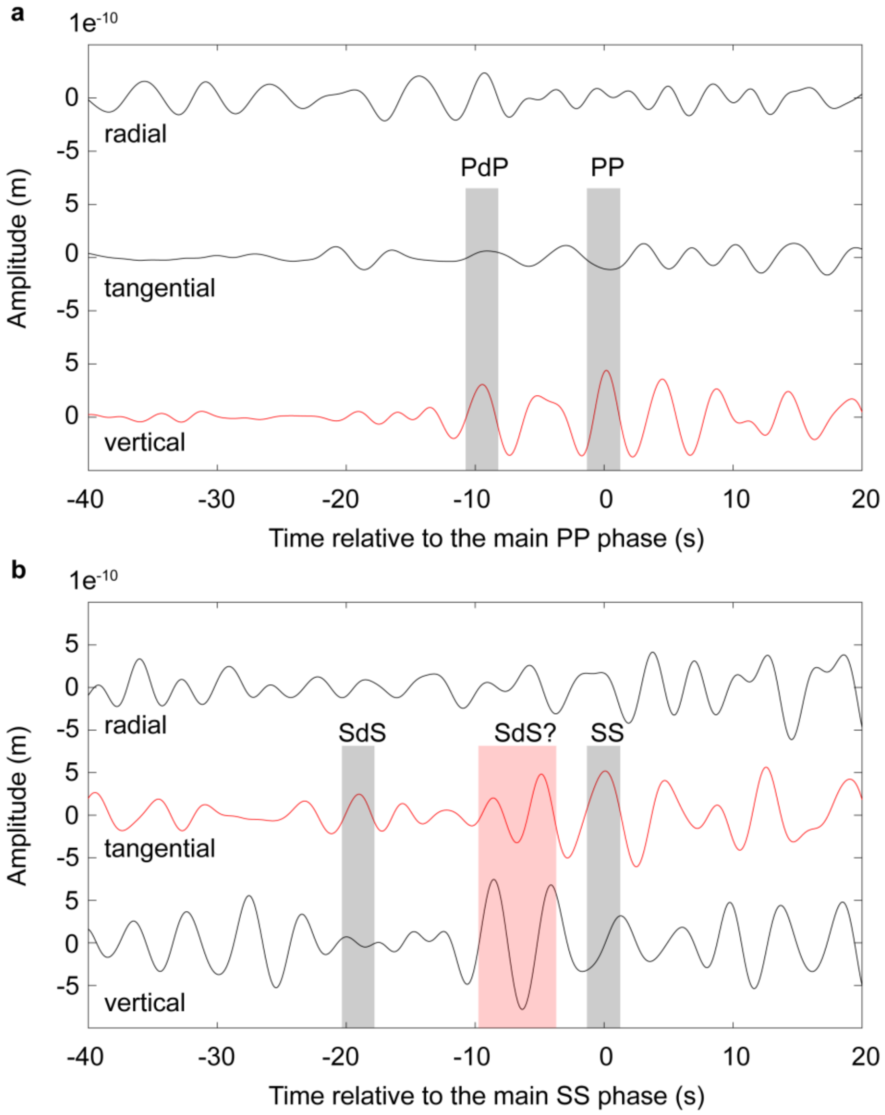

Supplementary Figure 2. Raw waveforms on three components.

(a) The three traces from the top to the bottom correspond to the raw displacement waveforms (near the PP arrival) filtered between 2.8 s and 6 s on the radial, tangential and vertical (in red, analyzed in this study) components, respectively. The shaded grey regions mark the PP phase and its precursor with almost no energy on the tangential component.

(b) The three traces from the top to the bottom correspond to the raw displacement waveforms (near the SS arrival) filtered between 2.8 s and 6 s on the radial, tangential (in red, analyzed in this study), and vertical components, respectively. The shaded grey regions mark the SS phase and its precursor with almost no energy on the vertical component. The signal in the shaded red region is less likely to be a SS precursor due to the large energy on the vertical component.

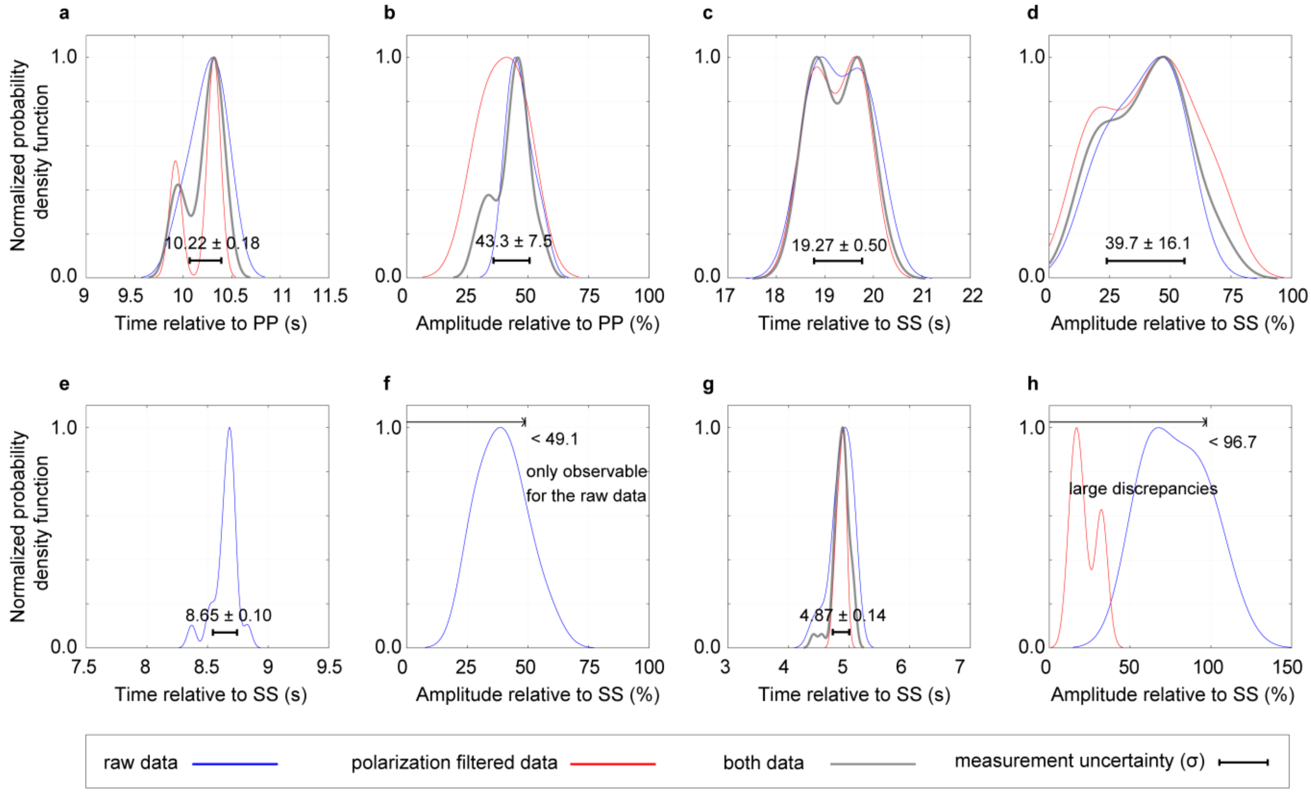

Supplementary Figure 3. Probability density functions for the differential arrival and amplitude ratio.

(a) Normalized probability density functions (PDFs) for the differential arrival between the main PP phase and PP precursor measured from Supplementary Fig. 1, based on the raw data (in blue), polarization-filtered data (in red), and the full dataset (i.e., both the raw data and the polarization-filtered data, in grey). The mean value and one standard deviation based on the full dataset are also annotated.

(b) Similar to (a), but for the amplitude ratio between the PP precursor and the main PP phase.

(c-d) Similar to (a-b), but for the SS precursor at ~ - 19 s.

(e-f) Similar to (a-b), but for another signal on the tangential component at ~ - 9 s. Note that this signal is only observable for the raw data in Supplementary Fig. 1. Therefore, in the inversion, we accept any amplitude ratio smaller than 49.1% (i.e., the mean value plus one standard deviation based on the raw data).

(g-h) Similar to (a-b), but for another signal on the tangential component at ~ - 5 s. Note that there is an inconsistency in the amplitude ratio between the raw data and the polarization-filtered waveform. Therefore, in the inversion, we accept any amplitude ratio smaller than 96.7% (i.e., the mean value plus one standard deviation based on the raw data).

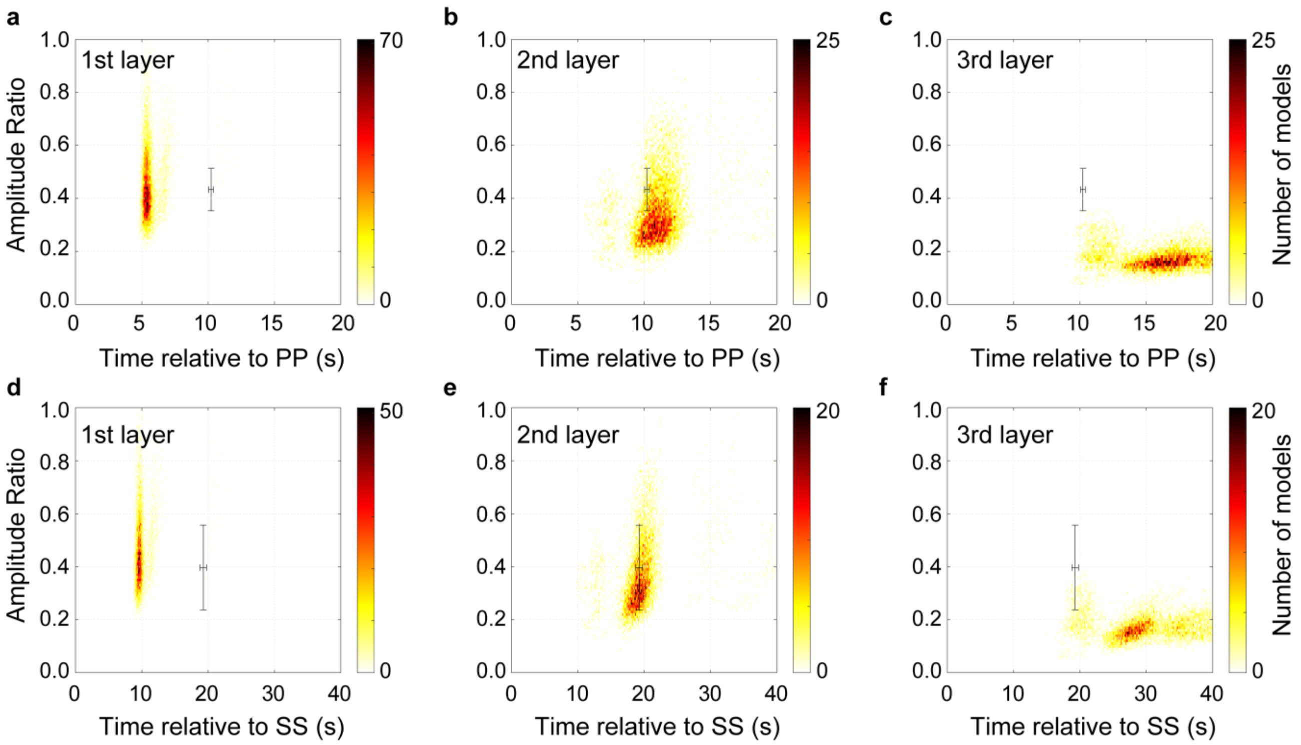

Supplementary Figure 4. Synthetic arrival and amplitude ratio for the receiver-function-derived models on Mars.

(a) Predicted differential arrival time and amplitude ratio between the PP phase and the PP precursor generated from the first (or top) layer in the receiver-function-derived models[1]. The horizontal and vertical error bars indicate the measurement uncertainties for the precursor analyzed in this study.

(b) Similar to (a), but for the second intra-crustal layer.

(c) Similar to (a), but for the third layer (i.e., Moho).

(d-f) Similar to (a-c), but for the SS waves.

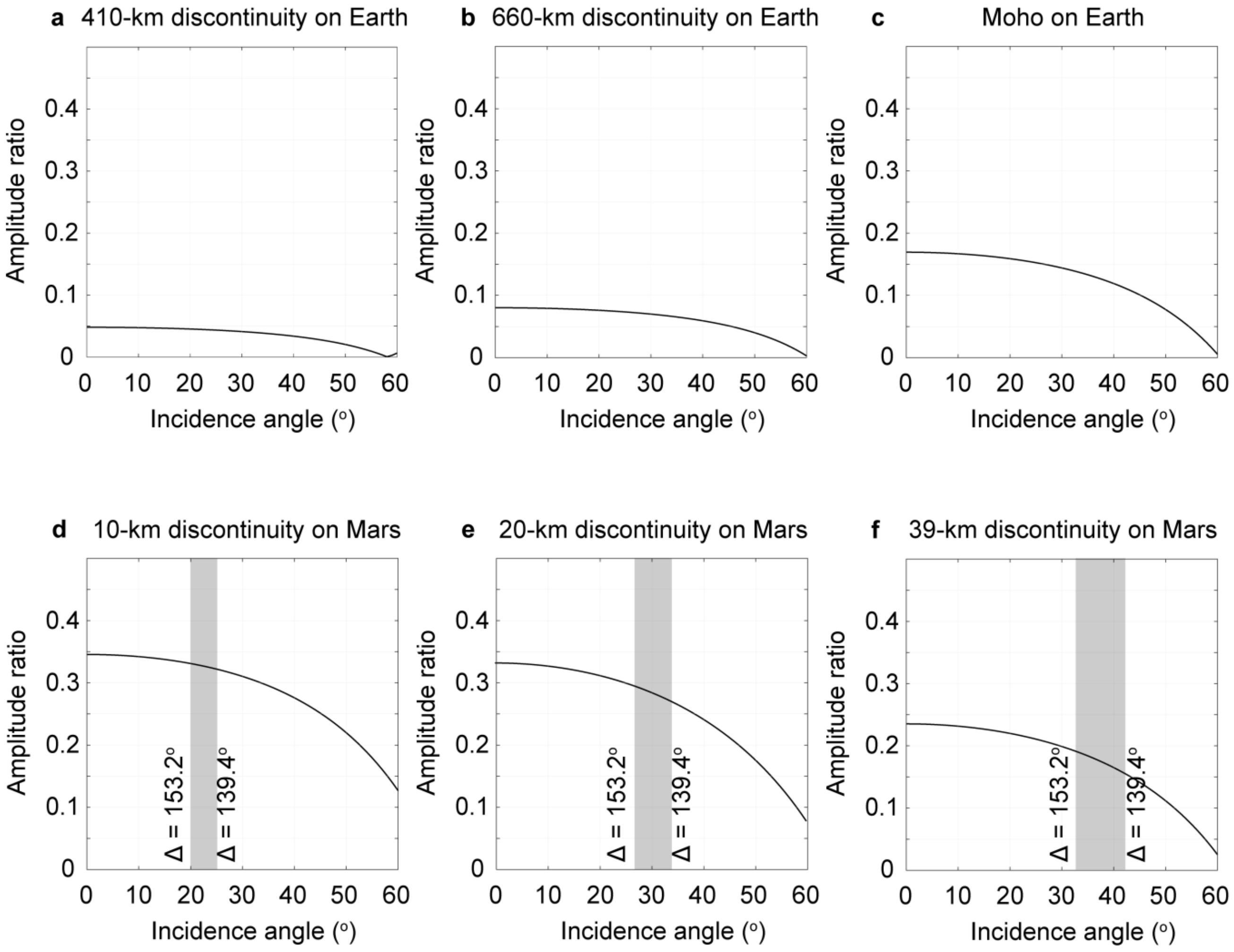

Supplementary Figure 5. Synthetic amplitude ratio from different discontinuities.

(a) For SS precursor generated from the 410-km discontinuity on Earth using the IASP91 model[2] for different incidence angles.

(b) For the 660-km discontinuity on Earth.

(c) For the Moho on Earth.

(d) For the first intra-crustal interface beneath the InSight lander on Mars, calculated using the mean value from 20,000 models in the receiver function study[1]. The shaded grey region marks the incidence angle range for the SS precursor from event S0976a based on the uncertainties in both the event location and mantle velocity models.

(e) For the second intra-crustal interface beneath the InSight lander on Mars.

(f) For the third interface (i.e., the Moho) beneath the InSight lander on Mars.

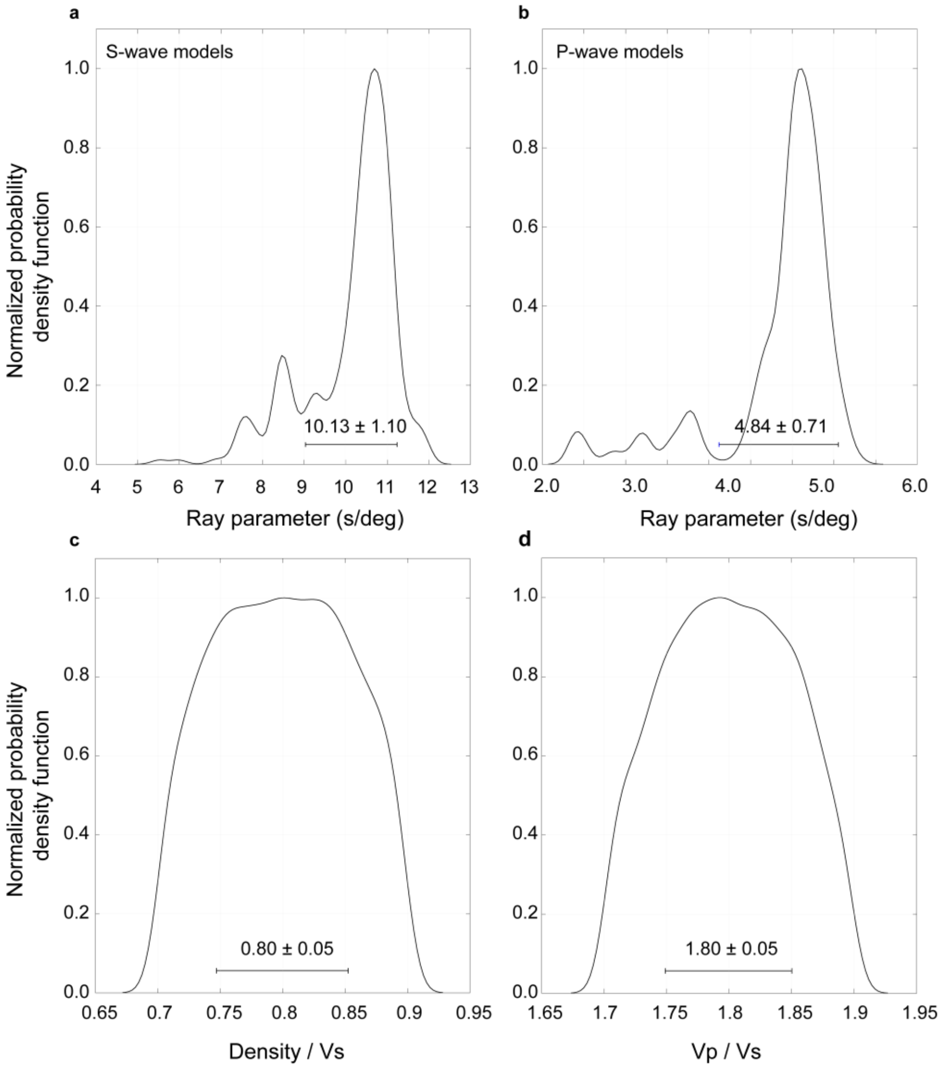

Supplementary Figure 6. Hyperparameters from other studies.

(a) Ray parameters for the S-waves from 100 models in Stähler et al. (2021) (ref.[3]) calculated using the Taup software[4]. The mean value and one standard deviation are also annotated.

(b) Similar to (a), but for the P-waves.

(c) The density-to-Vs ratio calculated from the 20,000 models in Knapmeyer-Endrun et al. (2021) (ref.[1]).

(d) Similar to (c), but for the Vp-to-Vs ratio.

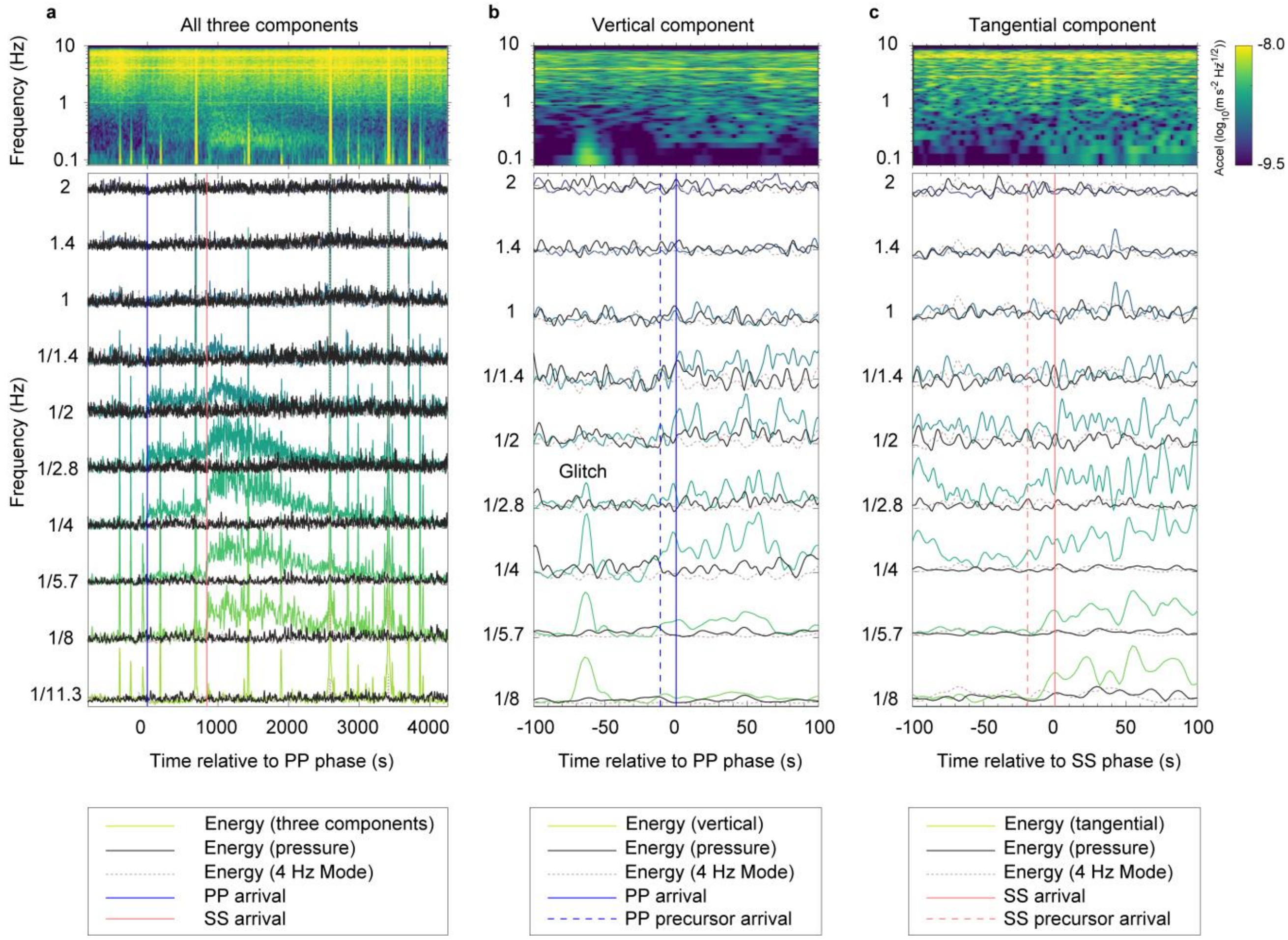

Supplementary Figure 7. Comodulation analysis.

(a) Comodulation analysis[5] for the total duration of the event S0976a. The top figure shows the spectrogram of the total signal power in all three axes (ZNE), calculated using 50-s long Hanning windows. In the bottom figure, the line traces show a comparison between the seismic, pressure, and estimated atmospheric power of the signal based on the 4 Hz lander resonance. The total seismic signal power (colored lines) and atmospheric pressure power (solid black) are calculated in half-octave windows centered from 1/11.3 to 2 Hz. The atmospheric signal power of the weather-sensitive lander resonance is calculated in the frequency range of 3.7 - 4.3 Hz (dotted grey). The power of the seismic and lander resonance signal is variance- and mean-matched to the pressure signal in the window before the P-wave arrival. Where the seismic line trace is above the atmospheric and lander-resonance line trace, the residual can be attributed to a seismic signal. PP- and SS-wave arrivals are marked by vertical blue and red lines, respectively.

(b) Comodulation analysis centered around a ± 100s window of the PP arrival on the vertical component. The main PP phase and PP precursor are marked by vertical solid blue and dashed blue lines, respectively.

(c) Comodulation analysis centered around a ± 100s window of the SS arrival on the tangential component. The main SS phase and SS precursor are marked by vertical solid red and dashed red lines, respectively.

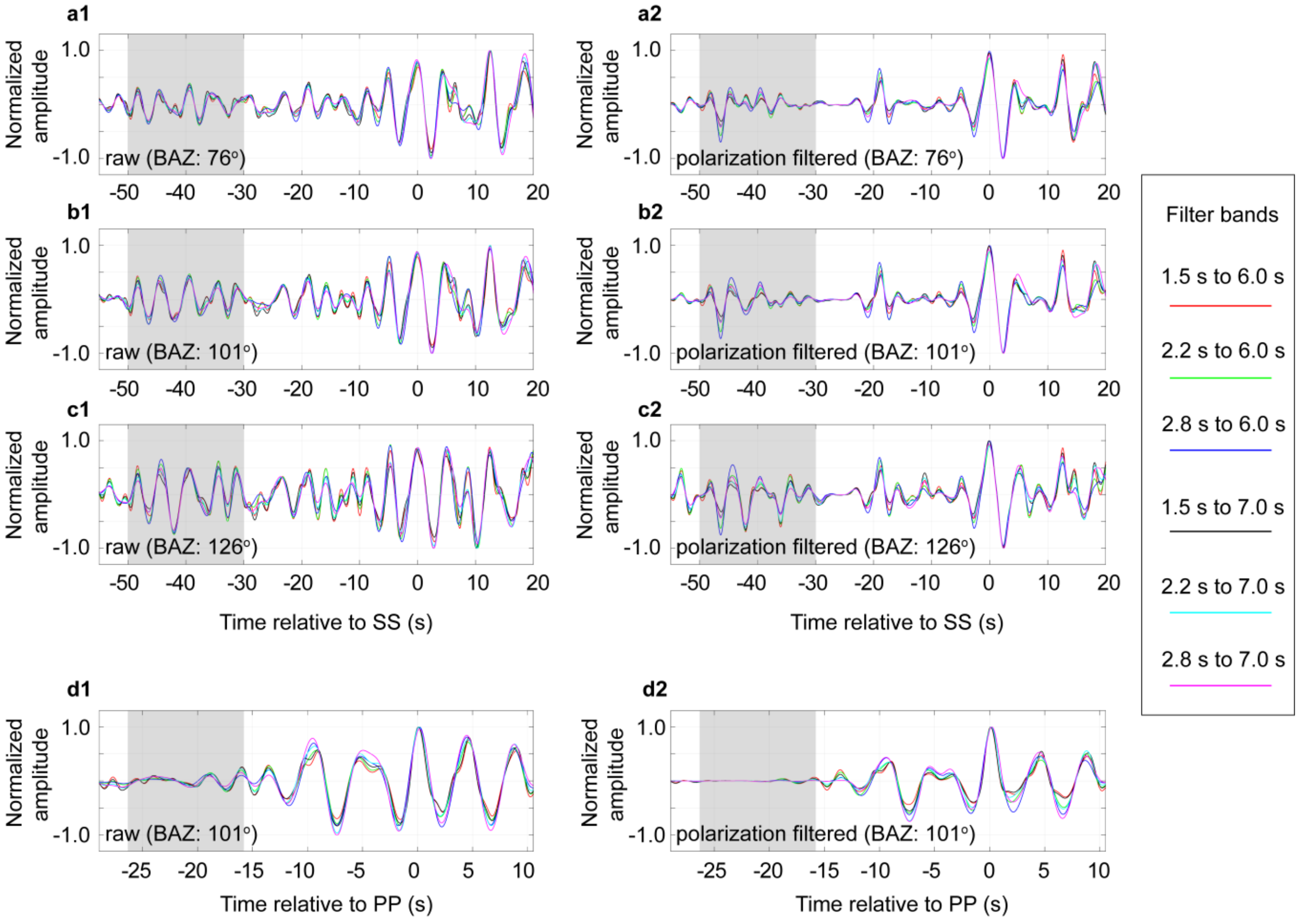

Supplementary Figure 8. Searching for earlier precursors.

(a1) Raw displacement data on the tangential component (based on a minimum back azimuth value of 76 degrees) filtered into six frequency bands, near the SS arrival.

(a2) Similar to (a1), but for the polarization-filtered data.

(b1-b2) Similar to (a1-a2) but based on a back azimuth value of 101 degrees.

(c1-c2) Similar to (a1-a2) but based on a maximum back azimuth value of 126 degrees.

(d1-d2) Similar to (a1-a2) but on the vertical component, near the PP arrival. Note the time axis for the PP phase is rescaled by 1.9 to better compare with the S-wave data.

The shaded grey region marks the searching window for earlier precursors.

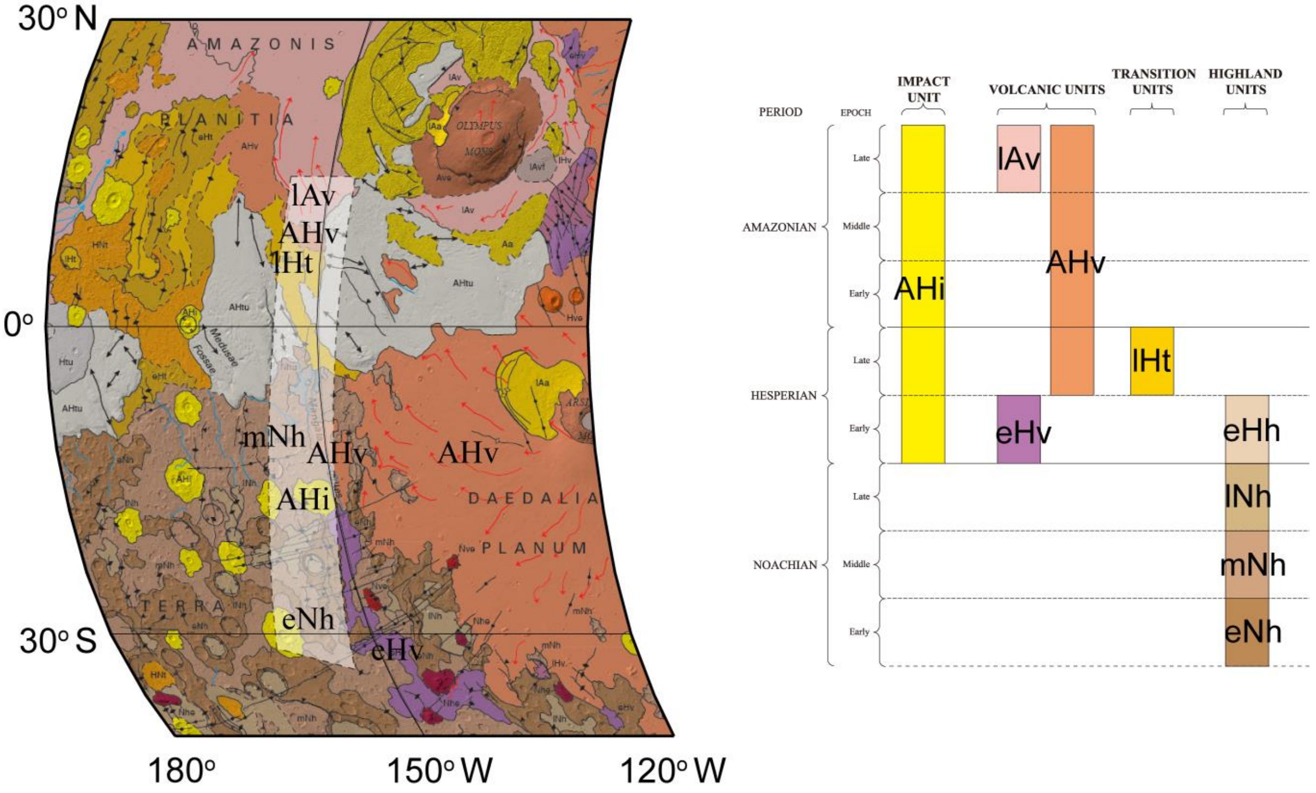

Supplementary Figure 9. Geological map near the bounce point.

Geological map, modified based on Tanaka et al. (2014) (ref.[6]), near the bounce point region (the shaded white region). The correlation of map units near the bounce point region is also modified based on Tanaka et al. (2014).

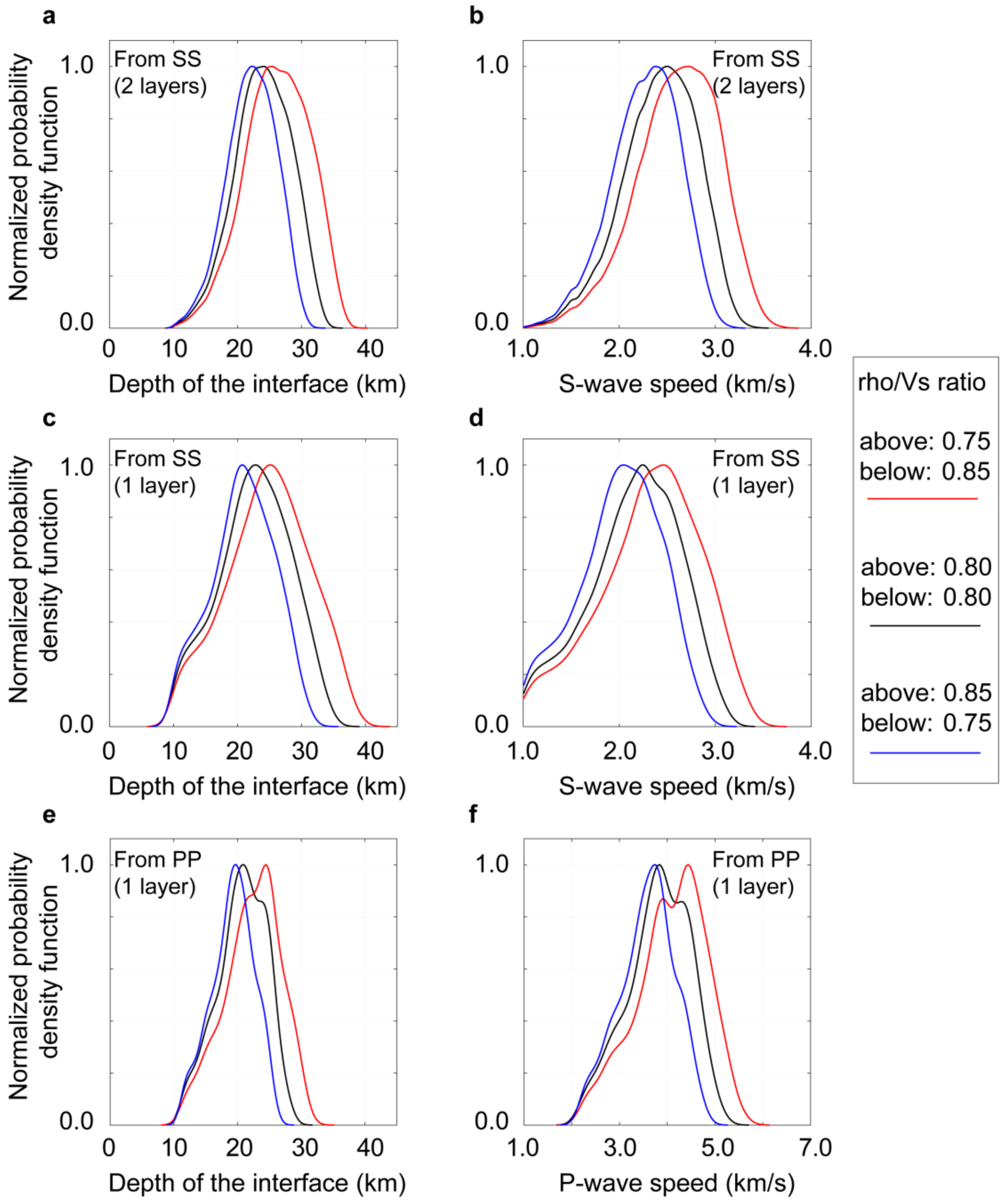

Supplementary Figure 10. Inversion results for different density-to-Vs values.

(a) The inverted depth distribution for the second interface (for the same two-layer SS inversion setting as in Fig. 4b) for the same density-to-Vs value of 0.8 in all layers is shown in black. The second inversion result with a smaller density-to-Vs ratio (0.75) above the interface and a larger density-to-Vs ratio (0.85) below the interface is shown in red. The third inversion result with a large ratio above and a small ratio below is shown in blue.

(b) Similar to (a), but for the inverted S-wave speed in the second layer.

(c-d) Similar to (a-b), but for the same one-layer SS inversion setting as in Fig. 4c.

(e-f) Similar to (a-b), but for the same one-layer PP inversion setting as in Fig. 4d.

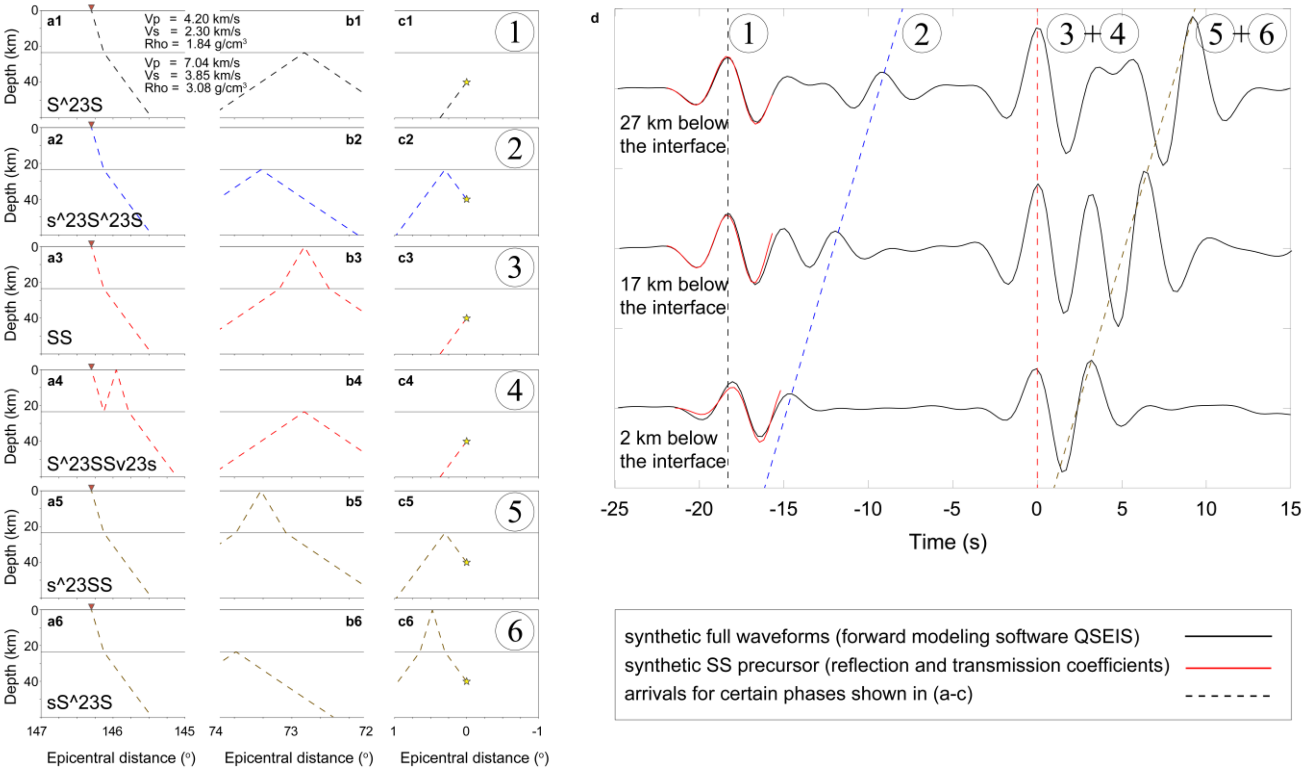

Supplementary Figure 11. Benchmark of the forward modeling approaches

(a1) Schematical seismic ray path for the SS precursor beneath the lander, at the bounce point (b1), and near the event (c1).

(a2-c2) Ray path for a depth phase (reflection off the intra-crustal interface) of the SS precursor.

(a3-c3) Ray path for the main SS phase.

(a4-c4) Ray path for a receiver-side reflection which arrives at the same time as the main SS phase.

(a5-c5) Ray path for a depth phase (reflection off the intra-crustal interface) for the main SS phase.

(a6-c6) Ray path for another depth phase (free surface reflection) of the SS precursor.

(d) Three synthetic waveforms (in black) with different focal depths (i.e., 2 km, 17 km, and 27 km below the intra-crustal interface at 23 km) are calculated using QSEIS software[7] with a 1-D Mars model (properties are shown in a1). The synthetic precursors modeled using the approach in this study (i.e., calculating the differential arrival and the amplitude ratio) are in red. The arrival time and amplitude ratio between these two modeling approaches are consistent when there is no significant interference from other phases. If there is interference (e.g., depth phases), since it shows up after both the main SS phase and its precursor, it does not significantly affect the phase identification. The travel time curves for the different seismic phases are annotated as dashed lines. Note that the receiver-side reflection (i.e., a4-c4) should be considered since the lander site and the bounce point share a similar interface depth of ~ 23 km.

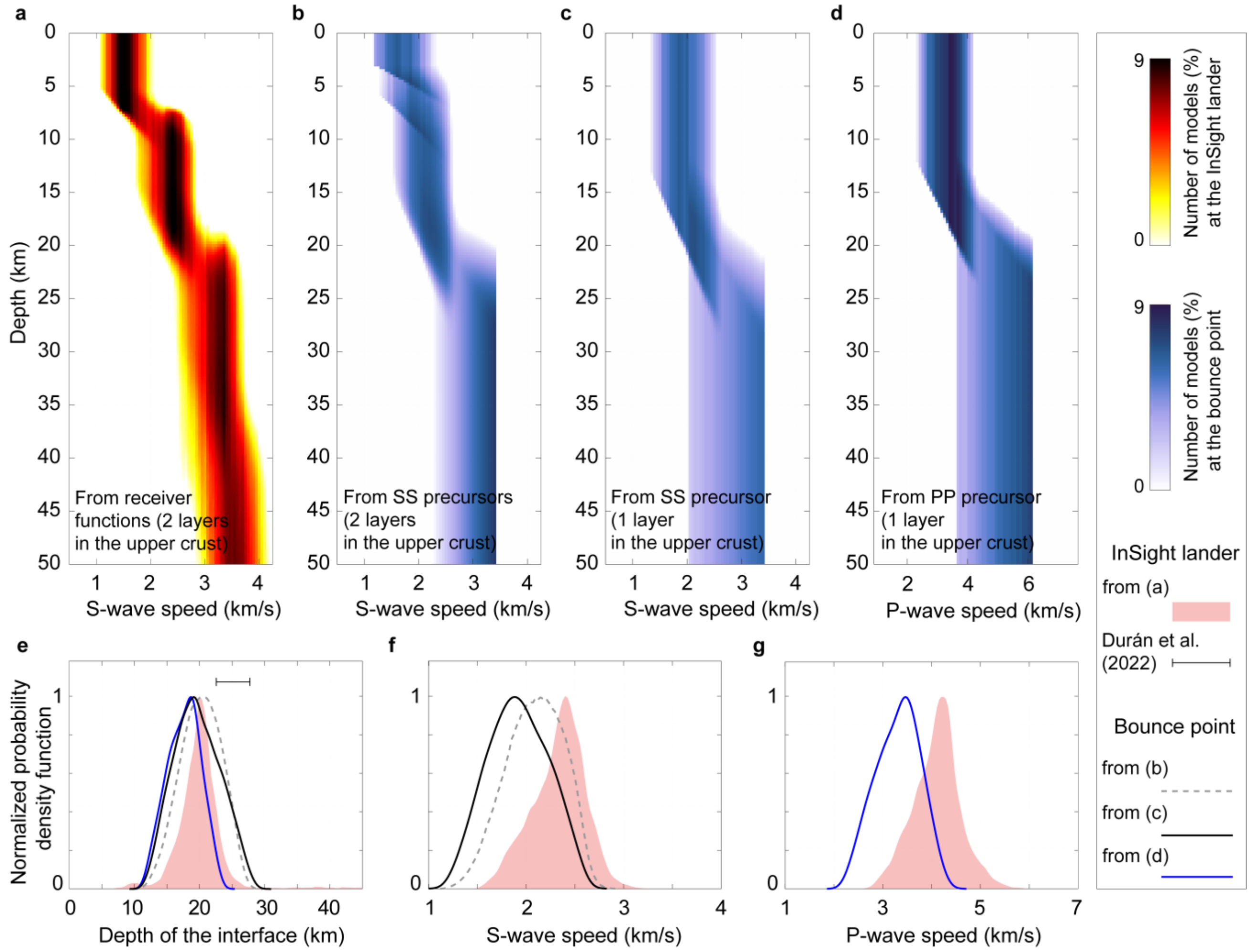

Supplementary Figure 12. Seismic velocity profiles from the inversion with updated priors.

(a) The three-layer crustal models beneath the InSight landing site from the receiver function study[1], with an intra-crustal interface at 20 ± 5 km. The color scale indicates the number of models.

(b) Inverted S-wave speed models at the bounce point with two layers in the upper crust from the SS precursors in this study.

(c) Similar to (b), but with one layer in the upper crust.

(d) Similar to (c), but for the P-wave speed with one layer in the upper crust.

(e) Normalized probability density functions for the depth of the intra-crustal interfaces beneath the landing site (in shaded red) from the receiver function study[8], and at the bounce point from the precursor constraints (in solid and dashed curves). The inferred interface depth at the landing site from another study[13] is also shown as a horizontal bar.

(f) Normalized probability density functions for the S-wave speed in the second layer beneath the lander (in shaded red), in the second layer for models in (b), and in the first layer for models in (c) at the bounce point.

(g) Similar to (f), but for the P-wave speed.

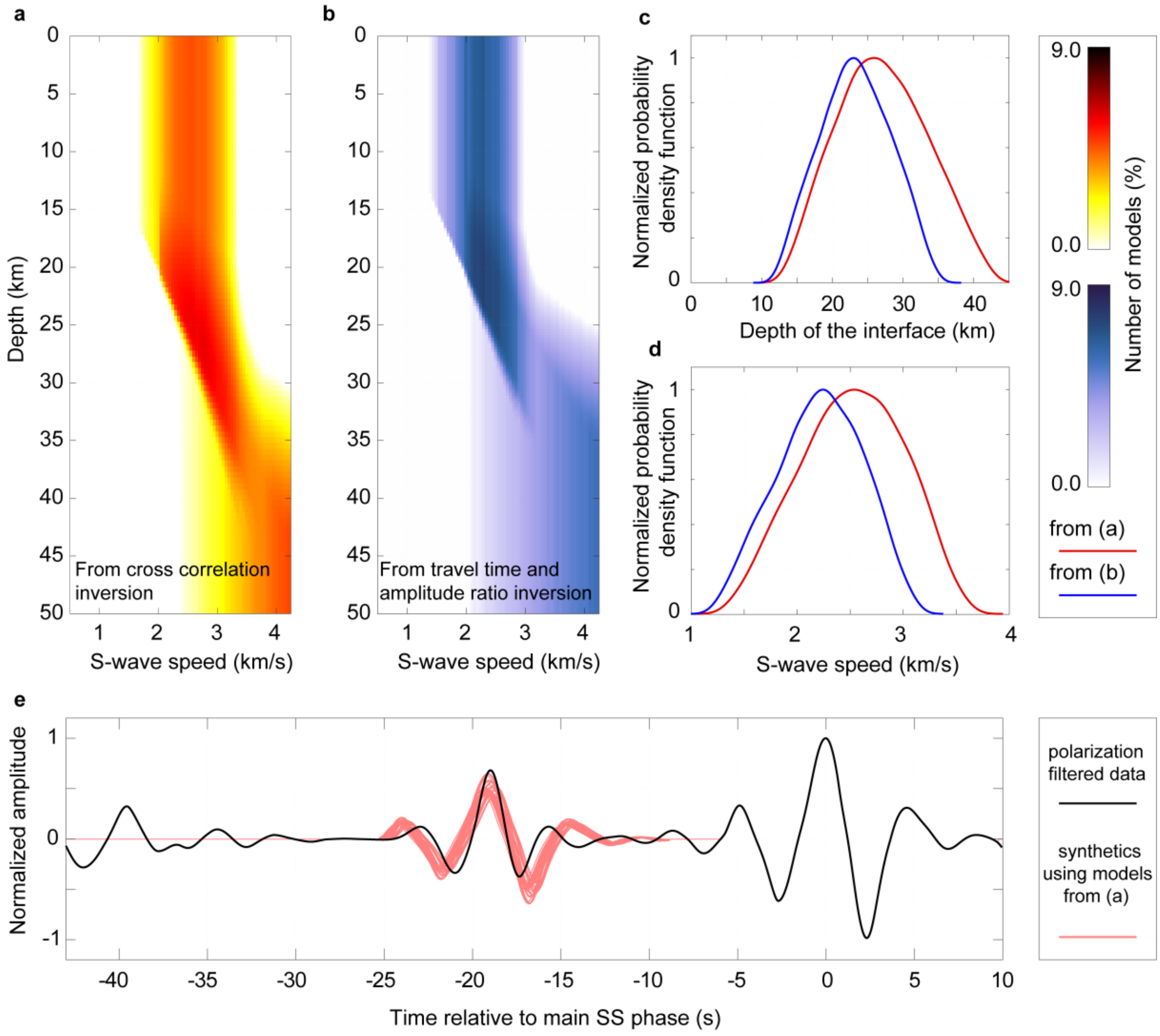

Supplementary Figure 13. Inversion results based on the waveform misfit.

(a) Acceptable models from the inversion based on the waveform misfit. The best top 5% models (i.e., with cross-correlation coefficients larger than 0.92) are viewed as acceptable models in this case.

(b) Acceptable models from the inversion based on the measured arrival time and amplitude ratio, i.e., Fig. 4c.

(c) Comparison of the interface depth between those two inversion approaches.

(d) Comparison of the S-wave speed in the intra-crustal layer between those two inversion approaches.

(e) Waveform comparison between the polarization-filtered data (filtered into 2.8 s to 6.0 s, in black) and the synthetic waveforms (the differential arrival and amplitude ratio are calculated using a subset of the acceptable models). Note that the left sidelobe of the SS phase is not picked in Supplementary Fig. 1 (due to the inconsistency between the raw data and the polarization-filtered data), but it is considered while calculating the cross-correlation coefficient.

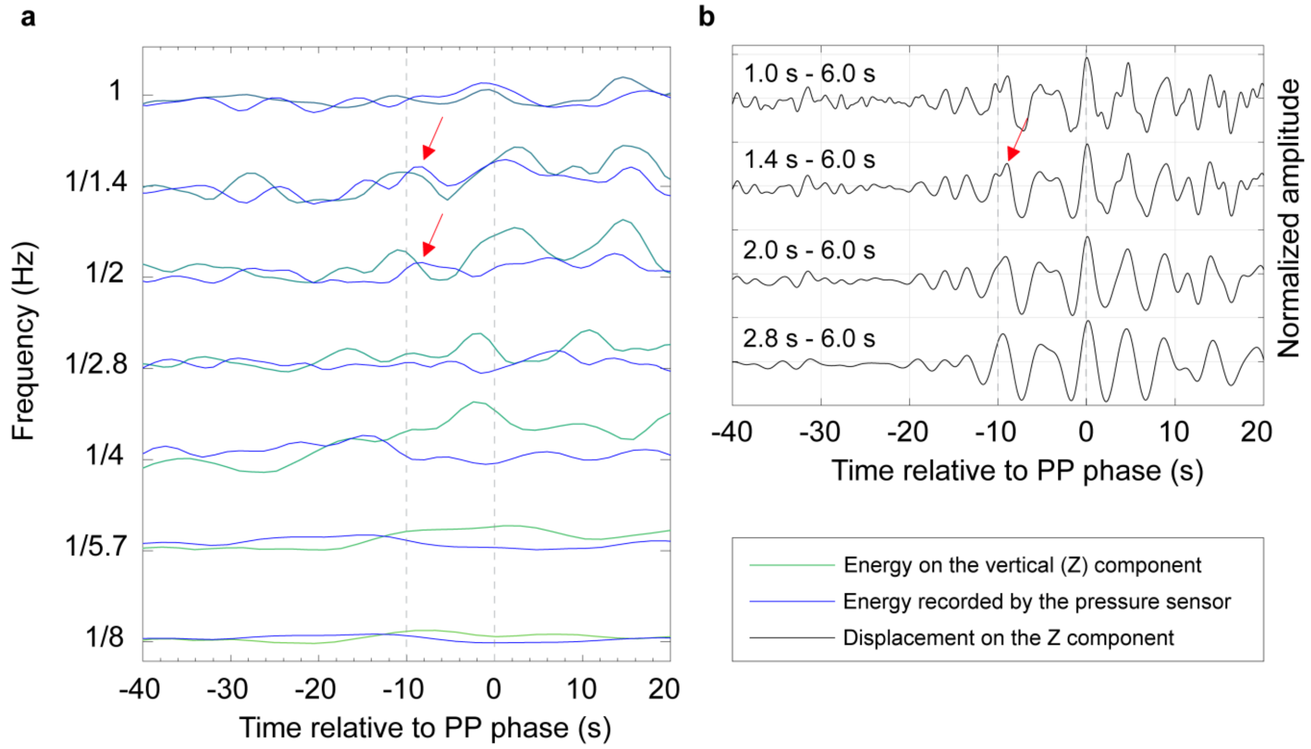

Supplementary Figure 14. Comodulation analysis for the PP precursor.

(a) The energy on the vertical component (in green) and recorded by the pressure sensor (in blue) filtered into different frequency bands (the center frequencies are annotated). The dashed vertical lines mark the location of the PP and PP precursor. The red arrows indicate the strong energy on the pressure data which is also observed in (b).

(b) Bandpass-filtered raw displacement waveforms on the vertical component. The red arrow points to the second positive peak in the precursor which is also observed in the pressure data in (a).

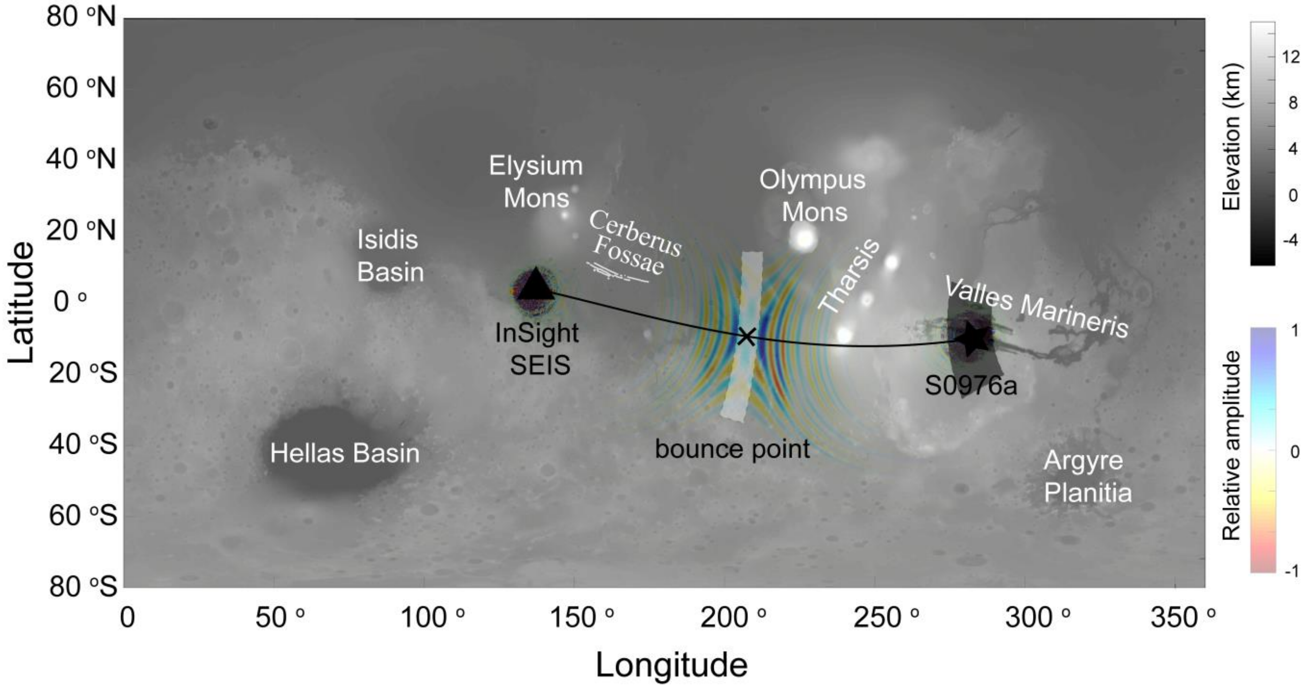

Supplementary Figure 15. Sensitivity kernel.

The sensitivity kernel of the PP phase was calculated at the shortest period of 5 s (see Methods section). The most sensitive region (i.e., the blue region of 12° wide and 20° long) is close to the bounce point, and its size is comparable to 1/3 of the uncertainty of the location of the bounce point (i.e., marked as the shaded white region). Note that due to the computation cost of the 3-D simulation, the sensitivity kernel here is only calculated at the shortest period of 5 s. For the seismic signal utilized in this study with higher frequencies (i.e., the half durations of PP and SS phases are less than 2 s and 3 s, respectively), the size of the corresponding sensitivity region will be smaller.

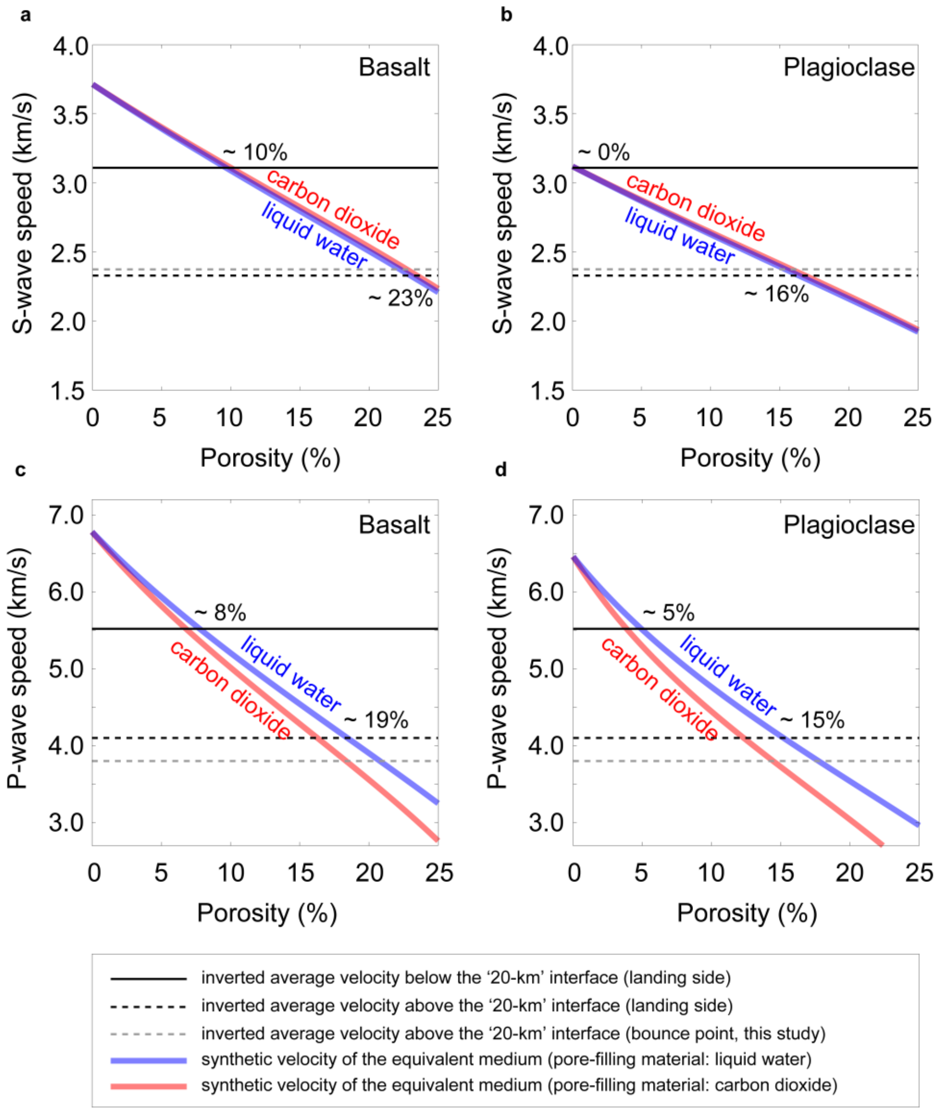

Supplementary Figure 16. Analysis of the porosity effects on the seismic wave velocities.

(a) S-wave speed of porous basalt with intrusions of carbon dioxide (in red) and liquid water (in blue) as a function of porosity (with the aspect ratio of 0.1). The solid black line represents the seismic velocity above the '20-km' discontinuity beneath the lander site[1]. The dashed black and grey lines indicate the seismic wave velocities above the pore closure depth at the lander site and the bounce point, respectively. The corresponding porosity estimations are also indicated (see Methods section).

(b) Similar to (a), but for plagioclase feldspar as the groundmass.

(c) Similar to (a), but for the P-wave velocity.

(d) Similar to (b), but for the P-wave velocity.

Table S1 Elastic properties of the materials in the porosity simulation

Material	Density (kg/m^3)	Bulk modulus (GPa)	Shear modulus (GPa)	References
Basalt	2,900	80.0	40.0	Christensen (1972); Heap (2019)
Plagioclase	2,630	75.6	25.6	Woeber et al. (1963)
Liquid water (20 °C)	1,000	2.2	0	Heap (2019)
CO_2 (20 °C)	1.8	0.0001	0	Heap (2019)

Supplementary Table 1. Elastic properties of the materials in the porosity simulation.
Density, bulk modulus, and shear modulus for the solid groundmass of the basalt, plagioclase, and the two pore fluids. Properties of basalt were taken from Christensen (1972) (ref.[9]) and Heap (2019) (ref.[10]). Properties of plagioclase were taken from Woeber et al. (1963) (ref.[11]). Properties of liquid water and CO2 (for a pressure of 0.1 MPa and a temperature of 20 °C) were taken from Heap (2019) (ref.[10]).

Supplementary References

1. Knapmeyer-Endrun, Brigitte, et al. "Thickness and structure of the martian crust from InSight seismic data." *Science* 373.6553 (2021): 438-443.
2. Kennett, B. L. N., and E. R. Engdahl. "Traveltimes for global earthquake location and phase identification." *Geophysical Journal International* 105.2 (1991): 429-465.
3. Stähler, S. C., Khan, A., Banerdt, W. B., Lognonné, P., Giardini, D., Ceylan, S., Drilleau, M., Duran, A. C., Garcia, R. F., Huang, Q., et al., 2021. Seismic detection of the martian core, Science, 373(6553), 443–448.
4. Crotwell, H. Philip, Thomas J. Owens, and Jeroen Ritsema. "The TauP Toolkit: Flexible seismic travel-time and ray-path utilities." Seismological Research Letters 70 (1999): 154-160.
5. Charalambous, Constantinos, et al. "A comodulation analysis of atmospheric energy injection into the ground motion at InSight, Mars." *Journal of Geophysical Research: Planets* 126.4 (2021): e2020JE006538.
6. Tanaka, K. L., Skinner, Jr., J. A., Dohm, J. M., Irwin III, R. P., Kolb, E. J., Fortezzo, C. M., Platz, T., Michael, G. G. and Hare, T. M. (2014) Geologic map of Mars: U. S. Geol. Surv. Sci. Invest. Map 3292, scale 1:20,000,000. https://dx.doi.org/10.3133/sim3292
7. Wang, Rongjiang. "A simple orthonormalization method for stable and efficient computation of Green's functions." *Bulletin of the Seismological Society of America* 89.3 (1999): 733-741.
8. Durán, C., et al. "Seismology on Mars: An analysis of direct, reflected, and converted seismic body waves with implications for interior structure." *Physics of the Earth and Planetary Interiors* 325 (2022): 106851.
9. Christensen, Nikolas I. "Compressional and shear wave velocities at pressures to 10 kilobars for basalts from the East Pacific Rise." *Geophysical Journal International* 28.5 (1972): 425-429.
10. Heap, Michael J. "P-and S-wave velocity of dry, water-saturated, and frozen basalt: Implications for the interpretation of Martian seismic data." *Icarus* 330 (2019): 11-15.
11. Woeber, A. F., Samuel Katz, and T. J. Ahrens. "Elasticity of selected rocks and minerals." *Geophysics* 28.4 (1963): 658-663.
