# Supplementary material for: Constraints on the martian crust away from the InSight landing site
Source: Nat Commun. 2022 Dec 26;13:7950. doi: 10.1038/s41467-022-35662-y (PMC9792460; doi:10.1038/s41467-022-35662-y)
Supplement: Supplementary file 3 — Description of Additional Supplementary Files [file 41467_2022_35662_MOESM3_ESM.pdf]

Description of Additional Supplementary Files

Supplementary Data 1: This folder contains the MATLAB codes for data processing and structure inversion.

The codes have been tested for MATLAB version R2021a with WINDOWS 10 system (19044.2130).

Those two codes could reproduce Figure 2 and Supplementary Figure 1 (the expected output)
Data_and_Pick_P.m: To plot the raw data and apply the polarization filter for the P-waves, together with the picked phases.
Data_and_Pick_P.m: To plot the raw data and apply the polarization filter for the S-waves, together with the picked phases.

Those three codes could reproduce Figure 4 (the expected output)
Inversion_P_1_Layer.m : Inversion code for the PP precursors, with one layer in the upper crust.
Inversion_S_1_Layer.m : Inversion code for the SS precursors, with one layer in the upper crust.
Inversion_S_2_Layers.m: Inversion code for the SS precursors, with two layers in the upper crust.

Those three codes could reproduce Supplementary Figure 16 (the expected output)
Basalt.m : Code for calculate the P- and S-wave speed of porous basalt with intrusions of carbon dioxide and liquid water as a function of porosity (with the aspect ratio of 0.1).
Plagioclase.m : Code for calculate the P- and S-wave speed of porous plagioclase with intrusions of carbon dioxide and liquid water as a function of porosity (with the aspect ratio of 0.1).

Data: Raw data, deglitched data (the codes can be run based on this data), and the instrument response.

util: Other related subroutines.
